# Deep learning approach for probabilistic pulmonary function estimation from chest X-ray and peak expiratory flow rate

**DOI:** 10.1038/s43856-026-01702-7

**Published:** 2026-06-09

**Authors:** Christoph Killing, Maximilian Wekerle, Jayne S. Sutherland, Mohammad Rassool, Lindsay Zurba, Olena Ivanova, Salome Charalambous, Celso Khosa, Robert S. Wallis, Michael Hoelscher, Claire Calderwood, Brian Allwood, Noemi Castelletti, Andrea Rachow

**Affiliations:** 1 Institute of Infectious Diseases and Tropical Medicine, LMU University Hospital, LMU Munich, Munich, Germany; 2 Vaccines and Immunity Theme, Medical Research Council Unit The Gambia at LSHTM, Fajara, The Gambia; 3 Clinical HIV Research Unit (CHRU), Wits Health Consortium (WHC), Health Science Research Office (HSRO), Faculty of Health Sciences, University of Witwatersrand, Johannesburg, South Africa; 4 Education for Health Africa, Durban, South Africa; 5 The Aurum Institute, Johannesburg, South Africa; 6 Instituto Nacional de Saúde, Marracuene, Mozambique; 7 German Centre for Infection Research (DZIF), Partner Site Munich, Munich, Germany; 8 Fraunhofer Institute, Immunology, Infection and Pandemic Research, Munich, Germany; 9 Helmholtz Zentrum München, German Research Center for Environmental Health (HMGU), Neuherberg, Germany; 10 Clinical Research Department, London School of Hygiene & Tropical Medicine, London, UK; 11 Division of Pulmonology, Department of Medicine, Stellenbosch University & Tygerberg Hospital, Cape Town, South Africa

**Keywords:** Medical imaging, Respiratory tract diseases

## Abstract

**Background:**

Spirometry remains the gold standard for assessing pulmonary function. Deep learning models have demonstrated potential for estimating measurements from chest X-rays (CXR). We aim to effectively address anatomical variability and integrate probabilistic reasoning to enhance estimation reliability near diagnostic thresholds.

**Methods:**

We developed a probabilistic machine learning framework to estimate the forced expiratory volume in the first second (FEV1) and the forced vital capacity (FVC) as measured through spirometry. Estimations use morphologically regularized CXRs and anthropometric-normalized peak expiratory flow rate (PEFR) as proxy for volumetric information unavailable in imaging. By estimating FEV1 and FVC z-scores, we decouple appearance from anatomic variability. We demonstrate our method on a multi-national cohort of pulmonary tuberculosis patients exhibiting diverse structural abnormalities and ventilatory impairments.

**Results:**

Using ensembles of neural networks, we analyze 982 CXR and spirometry pairs from 568 individuals. The best model achieves an area under curve (AUC) of 0.879 (FEV1; 99%CI 0.876, 0.881) and 0.853 (FVC; 99%CI 0.850, 0.856) in identifying moderate or severe lung-function impairment on a previously unseen test-set, signifying an AUC improvement of 0.144 (FEV1) and 0.118 (FVC) over previous methods. When allowing up to 10% of samples to remain unclassified due to uncertainty, AUC further rises to 0.894 (0.891, 0.896) and 0.857 (0.854, 0.860), respectively. Our method performs robustly across diverse impairment types and CXR pathologies.

**Conclusions:**

Our study shows that decoupling anatomical variability from functional assessment improves lung function estimation. Incorporation of probabilistic modeling improved diagnostic reliability around a decision threshold. Therefore, our system offers a promising approach to practical lung function estimation in settings where spirometry is unavailable.

## Plain language summary

Measuring how well the lungs work usually requires a breathing test called spirometry, which is not always available. Chest X-rays are widely used, but they do not directly show lung function. In this study, we aimed to estimate lung function from chest X-rays in a more reliable way. We used artificial intelligence to analyze X-rays together with basic body measurements and breathing flow information. We tested it on data from people with tuberculosis from different countries, who had several different types of lung damage. Our approach identified moderate to severe lung problems more accurately than previous methods. This could help doctors assess lung health in places where spirometry is hard to access and support better clinical decisions in the future.

## Abstract (web summary)

Killing et al. develop a probabilistic deep learning method to estimate lung function from chest X-rays using anatomical normalization and uncertainty modeling. The approach improves identification of moderate to severe impairment and provides more reliable results near diagnostic thresholds when spirometry is unavailable.

## Introduction

Spirometry is a cornerstone in the diagnosis and monitoring of chronic respiratory diseases (CRDs). This pulmonary function test produces key indicators of respiratory health, including forced expiratory volume in the first second (FEV1) and forced vital capacity (FVC). FEV1 is widely used to diagnose and quantify obstructive airway disease, which accounts for a significant proportion of the global morbidity and mortality burden associated with CRDs^1^. It strongly predicts mortality, hospitalization, and cardiovascular diseases in the general population and in patients with chronic obstructive airways disease, emphasizing the importance of lung function assessment for both diagnosis and prognostication^2,3^. FVC is an indicator of restrictive impairments to respiratory function, such as pulmonary fibrosis^4^.

However, spirometry is a technically challenging and time-consuming procedure that requires skilled operators and depends on patient effort. Additionally, commonly used guidelines for interpreting spirometry rely on qualitative measures, making results prone to operator bias^5^. Specialized personnel are required to ensure clinically accurate and reliable testing, restricting spirometry to well-controlled settings. This limits access to functional ventilatory impairment assessment, diagnosis, and treatment, especially in health resource-limited countries (HRLCs), where the burden of CRDs is highest^6^.

Post-tuberculosis lung disease, a commonly observed long-term sequela of pulmonary tuberculosis (TB), encompasses a range of functional impairments detectable by spirometry, spanning obstructive, restrictive, and mixed ventilatory defects. These often coexist with diverse structural abnormalities observable on chest X-ray (CXR), including cavities, loss of lung volume, and fibrosis^7,8^. To facilitate at-scale access to diagnosis and care for chronic respiratory diseases, effective methods for assessing respiratory abnormalities are needed.

In contrast to the complexities of spirometry, CXR is a patient effort-independent method for detecting lung pathology. It provides a complementary perspective of respiratory health and effectively reveals direct signs of acute and chronic disease affecting the airways or parenchymal structures of the lung. Advances in machine learning-powered computer-aided detection software have alleviated limitations caused by shortages of trained medical staff required to interpret radiological assessments, which enabled at-scale use of CXRs for detecting diseases like TB in HRLCs^9,10^.

Beyond detecting structural pathologies caused by CRDs, recent work has demonstrated that deep learning can be used to estimate lung function by generating point estimates for liter values of FEV1 and FVC from CXR images^11^. However, FEV1 and FVC measures are influenced by multiple factors. The resulting variability often places values within the related range of minimal clinically important differences^12^. However, these ranges are not necessarily associated with changes in lung morphology visible on CXRs. Additionally, patient-specific anatomical features directly observable on CXRs, such as thoracic dimensions, may confound estimates. Such complexities limit the applicability of deterministic machine learning approaches to estimate spirometry from raw CXR images^13^.

In this work, we develop a probabilistic framework for estimating pulmonary function and ventilatory impairment through FEV1 and FVC. We additionally investigate the effects of thoracic anthropometric normalization of CXR images and inclusion of peak expiratory flow rate (PEFR) measurements as a proxy orthogonal to imaging on the diagnostic performance. Our approach outperforms previous models, demonstrating more robust and clinically relevant assessments in a cohort of TB-survivors exhibiting diverse structural and functional impairments. The resulting framework presents a promising methodology for respiratory function assessment in patients with CRDs, especially in HRLCs.

## Methods

### Study design

This retrospective machine learning model development is based on the TB Sequel Cohort. It includes 1430 people with microbiologically confirmed active pulmonary TB at treatment initiation in The Gambia, Mozambique, Tanzania, and South Africa. Participants were recruited between 2017 and 2019 and prospectively followed up for at least 24 months. A detailed description of the study methodology and procedures can be found in the published study protocol^14^. We prepared this manuscript according to the TRIPOD guidelines for machine learning model development^15^.

### Data collection and quality control

Chest X-ray images were collected and assessed by on-site clinicians at diagnosis, after completion of treatment (month 6 after TB diagnosis), and at two or more years after diagnosis. At the study sites in The Gambia and South Africa, CXRs were collected digitally or on film with subsequent high-quality digitalization. The sites in Mozambique and Tanzania were not included in this work due to the low-quality digitalization of CXRs. Details can be found in the study flowchart in Supplementary Fig. 1.1.

Spirometry was collected at TB diagnosis, day 14, and months two, four, and six post TB treatment initiation, and subsequently every six months for up to five years. Following the American Thoracic Society (ATS) and European Respiratory Society (ERS) standards, spirometry was performed by regularly-trained and monitored medical staff using *ndd Medical Technologies* EasyOne-PC spirometers^5^. All FEV1 and FVC spirometry measurements were verified by an on-site clinical expert and an external Pan-African Thoracic Society-certified monitor.

Peak expiratory flow rate measurements were extracted from raw spirometric flow-volume loops and matched to CXRs by patient identifiers, date of birth, and date of visit. If multiple PEFR values were available per spirometry session, we selected the largest recorded value based on physiological considerations.

### Spirometry normalization

To ensure comparability between individuals, recorded pre-bronchodilator spirometry values need to be independent of demographic and anthropometric variations. We normalized FEV1 and FVC values for age, sex, and height through computing corresponding z-scores using the *other* standard from the Global Lung Function Initiative (GLI) reference equations (also compare Supplementary Table 1.1)^16,17^. Similarly, we express demographic-independent PEFR measurements as the ratio to their predicted values, which are computed using regression equations incorporating age, sex, and height^18^.

### Anthropometric chest X-ray equalization

In previous work, spirometric parameter estimation from CXRs relied on unprocessed images. As a consequence, individuals of larger build or deeper inspiration at the moment of recording occupied more pixels in the image than someone more petite or who inhaled less^11^. Therefore, the extent of the occupied image area could have directly informed an estimation system about the expected order of magnitude in pulmonary volumes. Furthermore, technical factors which may vary between centers, such as the distance from patient to X-ray tube and plate, can magnify thoracic dimensions due to divergent scatter. In this work, we normalize CXR images towards thoracic morphology. The system is therefore agnostic to a patient’s physical dimensions and learns to focus on the observable structural variations and severity of lung damage instead. Furthermore, to directly relate to a measure of functional impairment, we replace the estimation of liter values with normalized GLI z-scores.

**Fig. 1:**
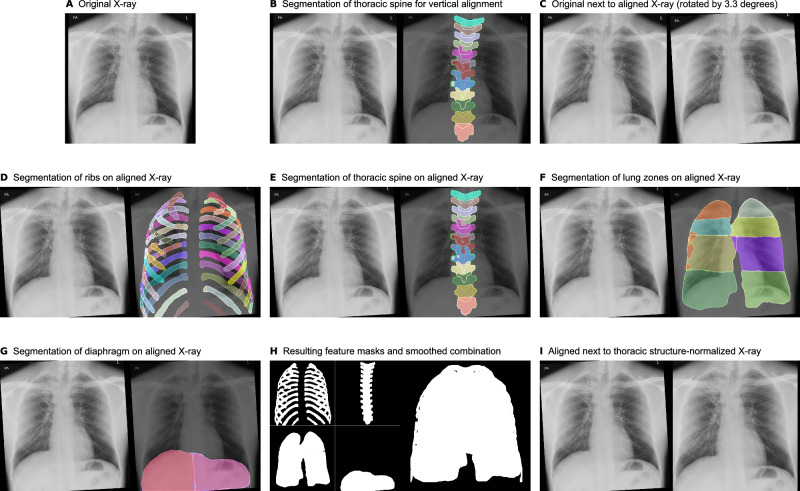
Normalization of chest X-ray (CXR) images to thoracic structure. **A** a representative sample of an original image. **B** segmentation of the thoracic spine by the Chest X-Ray Anatomy Segmentation package, subsequently used to rotate the CXR into a vertical alignment, of which the result is shown in (**C**). **D–G** segmentations of ribs, thoracic spine, lung zones and diaphragm based on the aligned X-ray images. **H** resulting masks and a smoothed combination of them, applied to the image to crop to consistent appearance throughout various patients. **I** (left) aligned CXR. **I** (right) resulting thoracic morphology-normalized image used by our system.

We utilized the *Chest XRay Anatomy Segmentation* package to generate semantic segmentation masks (SSM) for key anatomical structures found on our CXRs, including the thoracic spine, the ribs, the lungs, and the thoracic-abdominal border as defined by the diaphragm^19,20^. To standardize orientation, we align CXRs vertically through fitting an ellipse to the SSM of the thoracic spine and rotating the image until the major axis is vertical (Fig. 1A–C).

For anthropometric equalization, we reextract the SSM from the aligned image and create a combined mask of anatomical features (Fig. 1D–G). The relevant elements in the CXR image are identified as the difference of the skeletal elements minus the diaphragm in subsequent union with the lungs (Fig. 1H). We determine the extreme values along each dimension from this mask and use them to crop the image. To preserve anatomical proportions, we calculate the deltas between the extrema in both dimensions and use the larger one as the side length for a square cropping region (Fig. 1I). This results in anthropometric structure-normalized CXR images in which the lateral and upper outer boundaries are given by the skeletal structure. On the inferior end, artifacts in the abdominal cavity are removed while the costophrenic angle is preserved.

### Ground truth labeling and sample curation

Pulmonary function assessment outcomes from spirometry can vastly depend on participant effort and short-term changes to lung function, for instance, due to exposure to air pollution^21^. These variations can be more dynamic than changes to structural appearances. As spirometry readings from examinations fulfilling GLI standards can never overestimate the real value, images taken at the end of TB treatment were labeled with the highest value of FEV1 and FVC from within four months of the CXR date, where available. CXRs recorded afterwards were labeled the same way with spirometry results from within six months of the recording date. To accurately reflect the acute phase of TB disease, functional assessments at diagnosis were matched exclusively to CXRs from the same visit. We matched PEFR measurements to CXRs in the same way. The resulting CXR-spirometry pairs have reduced variability, as temporary disruptors to lung function evaluation were excluded where possible. However, the pairs are still subject to the aleatoric uncertainty arising from varying patient effort.

### Dataset composition

To allow our system to generalize to a wide range of individuals, all models were trained using k-fold cross-validation. We divided our data into training-validation and test sets in a 9:1 ratio, stratified by location, sex, and HIV status. Individual test estimates are subsequently combined using the arithmetic mean or, where indicated, a variance-weighted mean. We trained our models using 9-fold cross-validation by splitting the training-validation set in the same way in an 8:1 ratio for each fold. Since we split by patients, not by image, no patient is part of multiple sets. However, the number of CXRs can vary slightly between training-validation splits, as not all participants provided spirometry and CXR at every study visit.

### Model development

Convolutional neural networks have shown extraordinary performance in learning representations from labeled images. However, due to their capability to capture non-linear dependencies and absorb information, they require large amounts of training data to generalize well to previously unseen samples. Training time increases accordingly. In our dataset, as in most medical settings, the amount of data available is far less than what is required to train a network from randomly initialized parameters. To alleviate the burdens of both the long training times and comparably small datasets, we apply transfer-learning. Instead of randomly initializing a network with small weights and biases as commonly done when instantiating a new model, the network is initialized with weights and biases resulting from previous training runs on similar data. Subsequently, these network parameters are then adapted to the new task^22^.

**Fig. 2:**
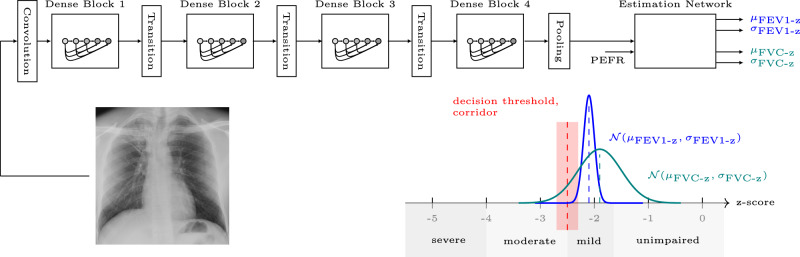
Overview of our system. We feed CXR images through a pretrained DensNet121 consisting of four dense blocks for feature extraction. Subsequently, we augment the image features with peak expiratory flow rate (PEFR) measures. Our fully connected estimation network then generates mean µ and scale σ of estimates for both FEV1 and FVC. These estimates are evaluated for their ability to distinguish unimpaired or mildly impaired lung function from moderately or severely impaired based on a decision threshold.

In this work, we use a pretrained DenseNet-121 convolutional neural network (CNN) as feature extractor^23^. Parameters are initialized from TorchXRayVision, an open-source library providing pre-trained models and publicly available CXR datasets^24^. We replace the final classification layer, which was pre-trained to distinguish pathologies, with a three-layer fully connected neural network (FCNN) for estimation. The feature vector extracted by the CNN from 224 by 224-pixel images is augmented with normalized PEFR values. The resulting low-dimensional representation is then fed through the FCNN, which estimates the mean and variance of a Normal distribution for FEV1 and FVC in the probabilistic use-case. During training, we freeze the weights of the first three dense blocks. The fourth block, as well as the FCNN, is adapted throughout 250 training epochs, minimizing the negative log-likelihood (NLL) of the observed data. An overview of our system is shown in Fig. 2, while further development details are reported in Supplementary Table 2.2.

When comparing to existing work on deterministic estimates, our model outputs only two continuous estimates: FEV1 and FVC, in liters or z-scores, depending on the setting and with or without inclusion of PEFR. We trained these benchmarking models using root mean-squared error (RMSE), previously identified as the most suitable error metric, and Huber loss, which was not previously considered but combines the advantages of mean-squared and mean-average error, two loss functions which were previously considered sub-par^11^.

We evaluated networks towards their ability to correctly distinguish mildly impaired or unimpaired pulmonary function from moderate or severe impairments, the latter of which are considered clinically relevant (Fig. 2). These groups are commonly differentiated by liter values exceeding 70% of the value predicted by the GLI reference equations or z-scores greater than −2·5, depicted in red^25^. Reported confidence intervals result from bootstrapping 1000 instances and computing the reported metrics for each (Supplementary Fig. 1.2).

In the probabilistic setting, three possible use cases for our model arise. First, we can estimate lung function and deduce corresponding impairments for all examined individuals without incorporating uncertainty, as in the deterministic case. Second, we might utilize the cumulative distribution function (CDF) of the Normal distribution specified by mean and variance. Through requiring a minimum share of the CDF to lie on either side of the decision boundary, we are effectively using it as a proxy for certainty. This, however, introduces an *“uncertain”* category, as some samples may not meet the required criteria, effectively transforming the binary classification into a three-class problem. Third, in combination with minimal CDF shares, instead of solely relying on a decision threshold, a zone of uncertainty allowing for measurement imperfection around it can be introduced. Subsequently, only model estimates fulfilling a minimal required share of the CDF are classified as impaired or unimpaired lung function (Supplementary Material, Section 3).

### Statistics and reproducibility

All experiments were run on a Ubuntu 24.04.1 machine with a NVIDIA GeForce RTX 4070 Ti GPU using Python 3.11.10, torch 2.5.1, and pytorch-lightning 2.4.0. We use PyTorch’s AdamW optimizer with a weight decay of 1e-5 and standard settings for the remaining parameters. We schedule the learning rate to reduce by a factor of 0.9 with a patience and cool-down of 2 up to a minimum value of 1e − 7.

### Human ethics and consent to participate

This work is based on the TB Sequel study for which ethical approvals were obtained previously. No additional data was collected or used in the work presented. Therefore, no additional IRB approvals were required for the work presented in this manuscript.

Ethical approvals for the study protocol and informed consent forms were obtained from the respective local and national Ethics Committees of the clinical study sites and from the Ethics Committee of the Ludwig-Maximilian University Hospital, Munich, Germany. They are registered with the

- Ethics Committee at the Ludwig Maximilian University Hospital, Munich under registration 786-16,
- República de Moçambique, Ministério da Saéde, Comité National de Bioética Para a Saúde under registration 200/CNBS/22,
- The Gambia Government / MRC Joint Ethics committee under registration 26487,
- Human Research Ethics Committee, University of the Witwatersand, Johannesburg under registration 161111, and
- The United Republic of Tanzania, Ministry of Health and Social Welfare, Mbeya Medical Research and Ethics Committee, under registration GB.152/377/01/10.

Prior to study enrollment, informed consent was obtained from all participants. All methods were carried out in accordance with relevant guidelines and regulations. The TB Sequel study was registered at ClinicalTrials.gov under number NCT03251196 (August 16, 2017).

## Results

Our analysis included 982 matched CXR images and spirometry evaluations from 568 individuals recovering from active pulmonary TB up to 60 months after initial diagnosis. A summary of the demographic and clinical characteristics as well as spirometry values split by training-validation and test set are shown in Table 1 and Table 2, respectively. Overall, 30·5% were female and 32·7% were HIV positive. The mean age at TB diagnosis was 37·1 years (95%CI: 35·1-39·1). Twelve percent of CXRs show cavities, 42·4% infiltrations, and 7·1% lobar volume loss across all study time points. The corresponding functional spirometry assessments had a mean of 2·5 liters for FEV1 (95%CI: 2·5, 2·6) and a mean of 3·2 liters for FVC (95%CI: 3·2, 3·3). Accounting for demographics, the corresponding z-scores according to the GLI *other* standard were −2·1 (−2·2, −1·8) and −1·9 (−2·0, −1·8), respectively^16^.

**Table 1:** Demographic and clinical characteristics and spirometry values of the training-validation dataset reported by study site in The Gambia (GMB) and South Africa (SA) and the study time point of diagnosis (M00), end of treatment (M06) and long-term follow-up including and after two years (M24 + )

		Overall	GMB - M00	GMB - M06	GMB - M24 +	SA - M00	SA - M06	SA - M24 +
**Training-Validation Set: n**		879	91	254	201	34	165	134
**Sex, n (%)**	**Female**	263 (29.9)	14 (15.4)	67 (26.4)	59 (29.4)	14 (41.2)	65 (39.4)	44 (32.8)
	**Male**	616 (70.1)	77 (84.6)	187 (73.6)	142 (70.6)	20 (58.8)	100 (60.6)	90 (67.2)
**HIV positive, n (%)**	**No**	616 (70.1)	86 (94.5)	235 (92.5)	186 (92.5)	6 (17.6)	54 (32.7)	49 (36.6)
	**Yes**	263 (29.9)	5 (5.5)	19 (7.5)	15 (7.5)	28 (82.4)	111 (67.3)	85 (63.4)
**Age at diagnosis [Y], median [Q1,Q3]**		34.4 [26.4,42.0]	34.2 [25.2,42.9]	31.6 [24.3,39.2]	31.3 [23.9,39.1]	41.0 [34.6,46.3]	37.0 [31.0,45.1]	37.8 [31.2,45.6]
**CXR Normal n (%) missing: 9**	**Yes**	262 (29.8)	0 (0.0)	49 (19.3)	108 (53.7)	2 (5.9)	42 (25.5)	61 (45.5)
	**No**	608 (69.2)	91 (100.0)	204 (80.3)	87 (43.3)	31 (91.2)	123 (74.5)	72 (53.7)
**CXR Ralph Score, median [Q1,Q3]**		5.0 [0.0,15.0]	50.0 [20.0,65.0]	5.0 [3.0,18.0]	0.0 [0.0,5.0]	30.0 [10.0,60.0]	5.0 [0.0,10.0]	2.0 [0.0,5.0]
**Cavities n (%)**	**No**	776 (88.3)	45 (49.5)	225 (88.6)	199 (99.0)	24 (70.6)	154 (93.3)	129 (96.3)
	**Yes**	103 (11.7)	46 (50.5)	29 (11.4)	2 (1.0)	10 (29.4)	11 (6.7)	5 (3.7)
**Infiltrations n (%)**	**No**	511 (58.1)	10 (11.0)	108 (42.5)	156 (77.6)	5 (14.7)	107 (64.8)	125 (93.3)
	**Yes**	368 (41.9)	81 (89.0)	146 (57.5)	45 (22.4)	29 (85.3)	58 (35.2)	9 (6.7)
**Lobar Volume Loss n (%)**	**No**	818 (93.1)	85 (93.4)	233 (91.7)	195 (97.0)	31 (91.2)	150 (90.9)	124 (92.5)
	**Yes**	61 (6.9)	6 (6.6)	21 (8.3)	6 (3.0)	3 (8.8)	15 (9.1)	10 (7.5)
**PEF [% predicted], median [Q1,Q3]**		70.7 [58.2,83.5]	57.9 [47.8,66.6]	68.0 [57.4,81.9]	71.8 [60.3,84.8]	62.5 [56.7,70.8]	75.4 [62.8,87.6]	77.7 [64.5,88.5]
**FEV1 [L], median [Q1,Q3]**		2.5 [2.0,3.0]	2.3 [1.7,2.8]	2.4 [2.0,2.9]	2.5 [2.1,3.1]	2.3 [2.1,2.7]	2.6 [2.2,3.2]	2.8 [2.3,3.4]
**FVC [L], median [Q1,Q3]**		3.2 [2.7,3.9]	2.8 [2.3,3.4]	3.0 [2.5,3.6]	3.2 [2.8,3.9]	3.2 [2.7,3.5]	3.4 [2.9,4.1]	3.6 [3.1,4.2]
**FEV1 (GLI predicted [L]), median [Q1,Q3]**		3.6 [3.0,4.0]	3.7 [3.2,4.1]	3.7 [3.2,4.1]	3.7 [3.1,4.1]	3.2 [2.7,3.8]	3.3 [2.8,3.8]	3.3 [2.9,3.8]
**FVC (GLI predicted [L]), median [Q1,Q3]**		4.3 [3.6,4.8]	4.5 [4.0,5.0]	4.5 [3.7,4.9]	4.4 [3.6,4.9]	4.0 [3.2,4.7]	4.0 [3.4,4.5]	4.1 [3.4,4.6]
**FEV1 (GLI z-score: other), median [Q1,Q3]**		−2.1 [−3.0,−1.2]	−2.9 [−3.6,−2.3]	−2.6 [−3.4,−1.8]	−2.2 [−2.9,−1.5]	−1.7 [−2.6,−1.0]	−1.5 [−2.3,−0.3]	−0.9 [−1.9,0.0]
**FVC (GLI z-score: other), median [Q1,Q3]**		−1.9 [−2.9,−1.0]	−3.1 [−4.0,−2.3]	−2.5 [−3.3,−1.7]	−2.0 [−2.8,−1.3]	−1.4 [−2.4,−0.6]	−1.1 [−2.0,−0.2]	−0.8 [−1.6,0.1]

**Table 2:** Demographic and clinical characteristics and spirometry values of the test dataset reported by study site in The Gambia (GMB) and South Africa (SA) and the study time point of diagnosis (M00), end of treatment (M06) and long-term follow-up including and after two years (M24 + )

		Overall	GMB - M00	GMB - M06	GMB - M24 +	SA - M00	SA - M06	SA - M24 +
**Test Set: n**		103	7	28	23	6	22	17
**Sex, n (%)**	**Female**	31 (30.1)	1 (14.3)	6 (21.4)	8 (34.8)	3 (50.0)	9 (40.9)	4 (23.5)
	**Male**	72 (69.9)	6 (85.7)	22 (78.6)	15 (65.2)	3 (50.0)	13 (59.1)	13 (76.5)
**HIV positive, n (%)**	**No**	68 (66.0)	7 (100.0)	27 (96.4)	21 (91.3)	0 (0.0)	7 (31.8)	6 (35.3)
	**Yes**	35 (34.0)	0 (0.0)	1 (3.6)	2 (8.7)	6 (100.0)	15 (68.2)	11 (64.7)
**Age at diagnosis [Y], median [Q1,Q3]**		35.5 [27.0,44.6]	29.0 [23.4,45.2]	30.5 [24.8,41.6]	34.1 [24.2,44.8]	34.4 [32.0,35.6]	35.9 [29.9,45.5]	36.2 [32.5,46.5]
**CXR Normal n (%) missing: 1**	**Yes**	33 (32.0)	0 (0.0)	3 (10.7)	15 (65.2)	0 (0.0)	5 (22.7)	10 (58.8)
	**No**	69 (67.0)	7 (100.0)	25 (89.3)	7 (30.4)	6 (100.0)	17 (77.3)	7 (41.2)
**CXR Ralph Score, median [Q1,Q3]**		5.0 [0.0,14.2]	65.0 [50.0,65.0]	6.5 [5.0,21.2]	0.0 [0.0,3.8]	12.5 [3.5,46.2]	5.0 [2.0,8.8]	0.0 [0.0,5.0]
**Cavities n (%)**	**No**	88 (85.4)	2 (28.6)	23 (82.1)	23 (100.0)	4 (66.7)	21 (95.5)	15 (88.2)
	**Yes**	15 (14.6)	5 (71.4)	5 (17.9)	0 (0.0)	2 (33.3)	1 (4.5)	2 (11.8)
**Infiltrations n (%)**	**No**	55 (53.4)	3 (42.9)	9 (32.1)	19 (82.6)	2 (33.3)	9 (40.9)	13 (76.5)
	**Yes**	48 (46.6)	4 (57.1)	19 (67.9)	4 (17.4)	4 (66.7)	13 (59.1)	4 (23.5)
**Lobar Volume Loss n (%)**	**No**	94 (91.3)	6 (85.7)	26 (92.9)	22 (95.7)	6 (100.0)	20 (90.9)	14 (82.4)
	**Yes**	9 (8.7)	1 (14.3)	2 (7.1)	1 (4.3)	0 (0.0)	2 (9.1)	3 (17.6)
**PEF [% predicted], median [Q1,Q3]**		69.8 [55.7,83.1]	59.3 [50.1,64.5]	56.9 [45.8,80.0]	64.4 [51.2,76.2]	65.0 [53.9,71.5]	84.7 [66.0,91.8]	76.2 [68.4,82.5]
**FEV1 [L], median [Q1,Q3]**		2.5 [2.0,2.8]	1.9 [1.9,2.5]	2.2 [1.8,2.6]	2.3 [1.9,2.6]	2.5 [1.8,2.7]	2.6 [2.4,2.9]	2.8 [2.6,3.3]
**FVC [L], median [Q1,Q3]**		3.1 [2.6,3.5]	2.7 [2.3,3.0]	2.9 [2.4,3.2]	3.1 [2.6,3.4]	2.8 [2.3,2.9]	3.3 [2.8,3.7]	3.9 [3.1,4.4]
**FEV1 (GLI predicted [L]), median [Q1,Q3]**		3.5 [3.1,3.9]	3.4 [3.2,3.9]	3.8 [3.2,4.1]	3.5 [3.0,4.1]	3.3 [3.2,3.4]	3.3 [3.0,3.7]	3.5 [3.2,3.7]
**FVC (GLI predicted [L]), median [Q1,Q3]**		4.2 [3.7,4.7]	4.2 [3.9,4.6]	4.6 [3.8,4.9]	4.2 [3.6,5.0]	3.8 [3.7,4.0]	3.9 [3.5,4.5]	4.3 [3.7,4.5]
**FEV1 (GLI z-score: other), median [Q1,Q3]**		−1.8 [−3.1,−1.2]	−3.7 [−4.0,−2.4]	−2.7 [−4.1,−1.7]	−1.9 [−3.3,−1.5]	−1.9 [−3.0,−1.2]	−1.7 [−1.9,−0.9]	−1.0 [−2.0,0.1]
**FVC (GLI z-score: other),median [Q1,Q3]**		−1.8 [−3.2,−0.7]	−3.7 [−4.3,−2.6]	−2.9 [−3.8,−1.4]	−2.0 [−3.2,−1.1]	−2.4 [−2.9,−2.0]	−1.5 [−2.0,−0.7]	−0.4 [−1.6,0.2]

### Normalized data improves estimates

Training networks using RMSE to estimate liter values from raw CXRs resulted in areas under the receiving operator characteristics (AUROCs) of 0·752 for FEV1 and 0·754 for FVC after conversion of estimates to binary impairment markers (compare Fig. 2). Normalizing data and using NLL as a loss function led to AUROCs of 0·844 for FEV1 and 0·826 for FVC, improving system performance over previously presented training using RMSE on raw data by 0·092 and 0·072 points, respectively. Improvements were driven by data representation rather than the loss function used (Table 3). Associated Bland-Altman plots for the underlying continuous estimates are shown in Supplementary Fig. 4.1 and Supplementary Table 4.1 while the stability of these results is confirmed in Supplementary Tables 5.1 and 5.2.

**Table 3:** Class-weighted AUROCs and 99% confidence intervals for several training settings

	Input Data Presentation	Input Modality	Estimation Target	Samples Classified	RMSE	Huber	NLL
**FEV1**	**raw**	**CXR**	**liters**	**all**	0.752 (0.748, 0.756)	0.748 (0.744, 0.752)	0.735 (0.731, 0.739)
		**CXR & PEFR**	**liters**	**all**	0.719 (0.714, 0.723)	0.700 (0.696, 0.704)	0.708 (0.704, 0.712)
	**normed**	**CXR**	**liters**	**all**	0.742 (0.738, 0.747)	0.738 (0.734, 0.742)	0.722 (0.717, 0.726)
			**z-score**		0.830 (0.827, 0.834)	0.853 (0.850, 0.856)	0.844 (0.840, 0.847)
		**CXR & PEFR**	**z-score**	**all**	0.865 (0.862, 0.867)	0.871 (0.868, 0.873)	0.879 (0.876, 0.881)
				**up to 10% uncertain**	NA	NA	0.894 (0.891, 0.896)
**FVC**	**raw**	**CXR**	**liters**	**all**	0.754 (0.750, 0.758)	0.739 (0.735, 0.744)	0.735 (0.731, 0.739)
		**CXR & PEFR**	**liters**	**all**	0.786 (0.782, 0.789)	0.785 (0.781, 0.788)	0.774 (0.770, 0.778)
	**normed**	**CXR**	**liters**	**all**	0.729 (0.725, 0.733)	0.732 (0.728, 0.736)	0.721 (0.717, 0.725)
			**z-score**		0.815 (0.811, 0.818)	0.831 (0.828, 0.834)	0.826 (0.822, 0.829)
		**CXR & PEFR**	**z-score**	**all**	0.839 (0.836, 0.842)	0.850 (0.847, 0.853)	0.853 (0.850, 0.856)
				**up to 10% uncertain**	NA	NA	0.857 (0.854, 0.860)

We evaluate the estimation performance of FEV1 and FVC represented as liter values or z-scores from CXR images (raw or morphology-normalized) and PEFR measurements. Our model was trained on continuous values with three loss functions, the RMSE, Huber loss and NLL of a Gaussian distribution. Detection of impairments is based on estimations of exceeding 70% of the value predicted according to GLI standards (in the case of liters) or a z-score greater than −2.5. Where all samples are classified, the arithmetic mean was used to combine ensemble votes for comparability. In the remainder of cases, a variance-weighted vote of the individual ensemble members was used. Wilcoxon signed-rank tests on underlying continuous estimation errors (per fold, combined using Fishers’ method) showed highly significant differences in results (*p* < 0.001) when comparing models trained with normed data and z-scores to the previously reported raw input and liter estimations.

### Peak expiratory flow rate further enhances estimates

Augmenting thoracic morphology-normalized CXRs by PEFR measures resulted in AUROCs of 0·879 for FEV1 and 0·853 for FVC when training with NLL (Table 3). This signifies an improvement of 0·144 and 0·118 for FEV1 and FVC, respectively, compared to using raw images without PEFR values to estimate liters. Through sensitivity analysis, we ensured that the improvements did not purely rely on PEFR measurements (Supplementary Fig. 1.2). We did not observe major performance differences between loss functions.

### Probabilistic methods improve classification accuracy and utility

**Fig. 3:**
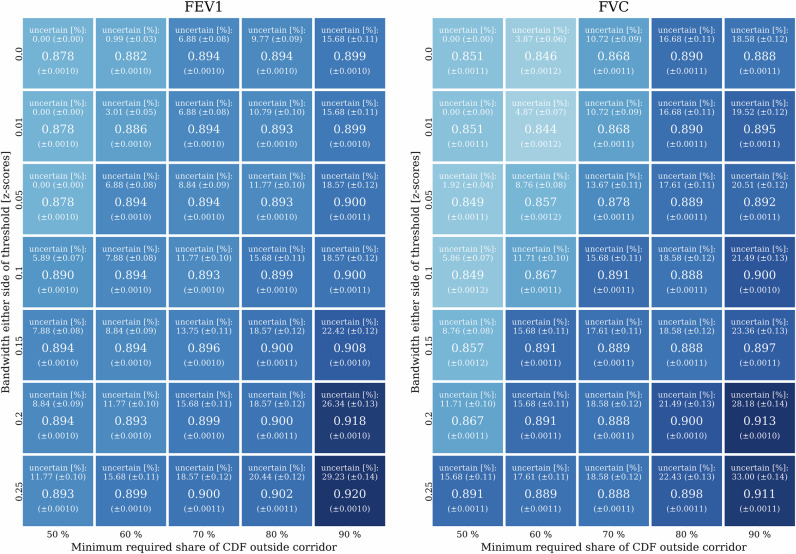
Heatmap of AUROC ( ± standard error) of variance-weighted estimates by spirometry metric (FEV1, FVC). Vertically, various bands around the z-score decision boundary of −2.5 are shown. The bandwidth of 0.25 to either side of the cutoff for FEV1 generates a zone of uncertainty with a width comparable to one minimal clinically relevant change (Supplementary Table 1.1). Horizontally, we increase the minimal share of the cumulative distribution function of the generated Normal distribution required outside of this decision corridor. We also report the share of datapoints for which no classification is possible, and therefore the network is uncertain about ( ± standard error) at the top of each box.

We further evaluate the network trained under NLL on normalized CXRs and PEFRs to estimate z-scores with the inclusion of uncertainty estimates. In Fig. 3, we horizontally increased the required probability mass from 50%, which allows classification of all samples, to 90%, which leaves a share of samples unclassified. Vertically, we addressed the variability of measures themselves by adding a band to either side of the z-score threshold of −2·5 (Fig. 2) up to a zone of uncertainty with a width comparable to one minimal clinically relevant change for FEV1 (Supplementary Table 1.1)^12^. The estimated value must be outside of this band to be classified. As the width and required probability mass outside this corridor increased, we observed an increase in AUROC accompanied by an expected increase in unclassified samples due to these stricter requirements. A higher share of samples remained unclassified for FVC than for FEV1. For direct comparisons in Table 3, allowing up to 10% of the samples to remain unclassified resulted in AUROCs of 0·894 for FEV1 and 0·857 for FVC.

### Results are stable across demographic subgroups

In addition to strong overall classification performance, models were stable across a range of demographic differences, structural abnormalities found on CXR, and functional impairment types identified through spirometry (Table 4). We defined functional impairments in line with recent literature^7^, using the latest recommended thresholds^25^. Our system was overall stable, with the lowest performance found on CXR images exhibiting loss of lobar volume. We show further results by site and over study time points in Supplementary Tables 6.1 and 6.2.

**Table 4:** Model performance metrics by chest X-ray pathology, spirometry impairment type, sex, and HIV status

		Parameter	AUC	F1	Precision	Recall
**Overall**	**(n** = **103)**	**FEV1**	0.878 (0.875-0.881)	0.750 (0.746-0.754)	0.766 (0.763-0.77)	0.753 (0.75-0.757)
		**FVC**	0.851 (0.849-0.854)	0.718 (0.714-0.721)	0.761 (0.758-0.765)	0.727 (0.724-0.731)
**Chest X-Ray Findings**	**Normal (n** = **33)**	**FEV1**	0.878 (0.873-0.884)	0.72 (0.711-0.728)	0.773 (0.766-0.78)	0.735 (0.727-0.742)
		**FVC**	0.865 (0.859-0.871)	0.626 (0.612-0.64)	0.691 (0.675-0.706)	0.668 (0.657-0.678)
	**Abnormal (n** = **69)**	**FEV1**	0.87 (0.867-0.873)	0.743 (0.739-0.747)	0.754 (0.75-0.758)	0.745 (0.741-0.75)
		**FVC**	0.838 (0.834-0.842)	0.714 (0.71-0.719)	0.752 (0.748-0.756)	0.723 (0.719-0.727)
**Chest X-Ray Cavities**	**Present (n** = **15)**	**FEV1**	0.910 (0.902-0.918)	0.781 (0.769-0.793)	0.802 (0.790-0.814)	0.788 (0.777-0.798)
		**FVC**	0.885 (0.876-0.893)	0.775 (0.762-0.787)	0.796 (0.783-0.808)	0.781 (0.770-0.793)
	**Absent (n** = **87)**	**FEV1**	0.852 (0.849-0.856)	0.708 (0.703-0.712)	0.737 (0.733-0.741)	0.715 (0.711-0.720)
		**FVC**	0.829 (0.826-0.833)	0.649 (0.644-0.655)	0.726 (0.722-0.730)	0.672 (0.668-0.677)
**Chest X-Ray Infiltrations**	**Present (n** = **48)**	**FEV1**	0.877 (0.873-0.881)	0.766 (0.761-0.771)	0.779 (0.774-0.784)	0.769 (0.764-0.774)
		**FVC**	0.827 (0.822-0.833)	0.695 (0.689-0.700)	0.730 (0.725-0.736)	0.704 (0.699-0.709)
	**Absent (n** = **54)**	**FEV1**	0.866 (0.862-0.870)	0.719 (0.713-0.725)	0.747 (0.742-0.752)	0.726 (0.721-0.732)
		**FVC**	0.895 (0.891-0.898)	0.722 (0.715-0.729)	0.778 (0.773-0.783)	0.735 (0.729-0.741)
**Chest X-Ray Loss of Lobar Volume**	**Present (n** = **9)**	**FEV1**	0.777 (0.759-0.795)	0.563 (0.547-0.579)	0.610 (0.591-0.629)	0.581 (0.565-0.596)
		**FVC**	0.701 (0.682-0.720)	0.665 (0.650-0.680)	0.702 (0.687-0.718)	0.678 (0.665-0.692)
	**Absent (n** = **93)**	**FEV1**	0.882 (0.879-0.884)	0.764 (0.760-0.768)	0.781 (0.777-0.784)	0.767 (0.763-0.771)
		**FVC**	0.855 (0.852-0.858)	0.712 (0.708-0.716)	0.765 (0.762-0.768)	0.724 (0.720-0.728)
**Functional Impairment**	**No impairment (n** = **33)**	**FEV1**	NA	0.934 (0.931-0.937)	1.0 (1.0-1.0)	0.878 (0.873-0.882)
		**FVC**	NA	0.984 (0.983-0.985)	1.0 (1.0-1.0)	0.969 (0.967-0.972)
	**restriction (n** = **41)**	**FEV1**	0.770 (0.764-0.777)	0.643 (0.637-0.649)	0.684 (0.678-0.69)	0.657 (0.651-0.662)
		**FVC**	0.780 (0.772-0.788)	0.542 (0.536-0.548)	0.585 (0.577-0.593)	0.564 (0.558-0.570)
	**obstruction (n** = **7)**	**FEV1**	0.830 (0.813-0.847)	0.515 (0.490-0.539)	0.566 (0.538-0.594)	0.570 (0.551-0.589)
		**FVC**	NA	1.0 (1.0-1.0)	1.0 (1.0-1.0)	1.0 (1.0-1.0)
	**mixed (n** = **16)**	**FEV1**	NA	0.967 (0.965-0.97)	1.0 (1.0-1.0)	0.939 (0.934-0.943)
		**FVC**	0.936 (0.93-0.941)	0.71 (0.695-0.725)	0.817 (0.807-0.828)	0.747 (0.736-0.758)
**Sex**	**Female (n** = **31)**	**FEV1**	0.946 (0.942-0.95)	0.779 (0.768-0.79)	0.794 (0.783-0.806)	0.785 (0.775-0.795)
		**FVC**	0.909 (0.902-0.916)	0.85 (0.839-0.862)	0.881 (0.871-0.891)	0.859 (0.850-0.869)
	**Male (n** = **72)**	**FEV1**	0.871 (0.867-0.874)	0.761 (0.757-0.765)	0.786 (0.782-0.790)	0.766 (0.762-0.770)
		**FVC**	0.843 (0.840-0.847)	0.687 (0.683-0.691)	0.724 (0.719-0.728)	0.697 (0.693-0.701)
**HIV Status**	**Positive (n** = **35)**	**FEV1**	0.955 (0.952-0.958)	0.804 (0.794-0.815)	0.851 (0.843-0.859)	0.816 (0.807-0.825)
		**FVC**	0.955 (0.952-0.958)	0.749 (0.739-0.759)	0.817 (0.809-0.824)	0.767 (0.759-0.775)
	**Negative (n** = **68)**	**FEV1**	0.814 (0.810-0.818)	0.706 (0.701-0.710)	0.713 (0.709-0.718)	0.708 (0.703-0.712)
		**FVC**	0.784 (0.779-0.788)	0.695 (0.690-0.699)	0.728 (0.723-0.732)	0.703 (0.699-0.707)

We report area under curve (AUC) and weighted F1-score as well as Precision and Recall in distinguishing mildly or unimpaired function tests from moderately or severely impaired ones for all samples. For some functional impairments, an area under the receiving operator characteristics cannot be computed as the phenotype definition requests unimpaired measurements only.

## Discussion

In this study, we presented a deep learning system capable of assessing lung function from CXRs. We showed that directly estimating spirometry parameters from raw CXRs proved insufficient in a heterogeneous population of TB survivors with diverse structural and functional abnormalities (Table 3). Normalizing both CXRs and spirometry measures improved estimation accuracy. However, CXRs, conventionally taken at full inspiration, may have limitations in capturing certain aspects of pulmonary function. The resulting dimensional mismatch in estimating a volume from an image was previously noted as an open challenge^13^. We propose the augmentation of morphology-normalized radiography with PEFR measurements to overcome this limitation of imaging. PEFR can correlate to pathology in the smaller airways, which may be difficult to detect on CXR but is likely to contribute to reduced lung function, particularly in FEV1^26^. The resulting improvements from this addition of a notion of maximal flow in combination with the radiological dimensions suggest PEFR is a viable marker orthogonal to imaging.

While spirometry remains the gold standard for pulmonary function testing, it is unavailable in many HRLCs. This limits management of respiratory diseases, especially in areas with high incidence rates of TB. In contrast to full spirometry, where various qualitative or quantitative standards need to be met, PEFR recordings are not subject to such strict requirements, although being still effort dependent. However, no definite consensus has been reached on the comparability of resistance-based peak flow meters and PEFR extracted from spirometry as used in this work^27^ The evidence shows some meters slightly over- and others slightly underestimate values obtained from spirometry, yet PEFR meters have previously been considered non-inferior in ambulatory settings^28^ Early work on benchmarking recently evolving digital turbine-based PEFR meters suggests a strong correlation to PEFR from spirometers^29^ An integration of these modern digital turbine-based peak-flow meters with our CXR-based analysis through a neural network could provide a viable alternative to traditional spirometry.

Despite being less common in health-care applications, the probabilistic neural networks presented here provide an estimate of uncertainty associated with a specific measure^30^ This is particularly useful when producing binary results, such as the diagnosis of impaired lung function using a continuous estimate, where results close to the decision boundary are subject to diagnostic uncertainty. For instance, in Fig. 2, where the estimate for FEV1 has a higher share of the probability mass outside the zone of uncertainty than the corresponding FVC estimate, is shown. This advancement over previous work allows for better control over misclassification tolerance at decision thresholds and, practically, may lead to more accurate diagnosis in patients with lung-function impairments^11^

Potential applications of our system might go beyond its integration in TB active case-finding approaches using CXR as a screening tool for TB and associated lung damage. In clinical settings where spirometry is unavailable, our system could be valuable for both lung function testing and risk assessments in patients screened for CRDs, as it was shown that lung function is predictive of long-term cardio-respiratory morbidity and mortality^2^ Our approach can also potentially be used as triage tool in settings where spirometry is available but limited in capacity as it can generate estimates and flag patients in need of spirometric testing while allowing the remainder to omit spirometry. Thereby, it optimizes resource utilization while maintaining a high degree of diagnostic confidence. Furthermore, since the underlying estimation network is continuous, our system may be capable of moving beyond binary classification to multi-class reporting, allowing for further refinement in the presented and further use-cases.

Despite the advances presented, our study has limitations. Regarding the underlying data, we focus on the development and evaluation of a prediction model under controlled research conditions using curated retrospective data. All input variables were preprocessed and quality-controlled before model training and evaluation. The assessment and handling of missing or poor-quality input data at deployment is outside the scope of this work and was therefore not addressed. On the methodological end, we did not assess the epistemic uncertainty of our model regarding its own parameters, which could be addressed in future work using a fully Bayesian approach. With respect to clinical application, our dataset was collected from TB-survivors at two study clinics in sub-Saharan Africa and includes patients with significant structural lung abnormalities. While our system performed similarly well across clinical subgroups and our data captures all major spirometry impairment types, the generalizability of our results to other clinical use-cases and further CRDs, some of which may exhibit similarly relevant structural and functional abnormalities, remains unclear. Further validation across diverse populations and healthcare systems remains essential to realize the full potential of this technology in clinical practice. In future work, besides extending the databases to more countries and health systems to exclude any ascertainment biases, the influence of lateral CXRs on the estimation should be evaluated.

In conclusion, we introduced a deep learning framework for lung function assessment relying on affordable and available clinical diagnostic tools. Our system enables functional lung-health assessment where no spirometry is available and can optimize the use of spirometry capacity where it is available. Our findings indicate that combining morphology-normalized radiography with PEFR measurements improves pulmonary function estimation, offering a feasible surrogate in these environments. Looking ahead, ensuring equitable access to these data-driven innovations, particularly in low-resource settings, will be critical for maximizing their global impact.

## Supplementary information

Supplemental Information

Description of Additional Supplementary files

Supplementary Data 1

Supplementary Data 2

## Data Availability

All source data underlying Figs. 1 and 2 are contained within the figures themselves. Raw model outputs are provided in Supplementary Data 2. Trained network weights and chest X-ray images cannot be shared to protect patient privacy. Instead, cohort summary statistics are provided in Tables 1 and 2. Together with the corresponding ground-truth data, Supplementary Data 2 can be processed to reproduce Fig. 3 and Tables 3 and 4 using the implementation provided in the cited code repository. Requests for additional information may be directed to Andrea Rachow (rachow[at]lrz[dot]uni-muenchen[dot]de).

## Code availability

The implementation is open sourced^31^.
