# Supplementary material for: Deep learning approach for probabilistic pulmonary function estimation from chest X-ray and peak expiratory flow rate
Source: Commun Med (Lond). 2026 Jun 9;6:330. doi: 10.1038/s43856-026-01702-7 (PMC13249966; doi:10.1038/s43856-026-01702-7)
Supplement: Supplementary file 1 — Supplemental Information [file 43856_2026_1702_MOESM1_ESM.pdf]

Online Data Supplement

Deep learning approach for probabilistic pulmonary function estimation from chest X-ray and peak expiratory flow rate

Christoph Killing[1], Maximilian Wekerle[1], Jayne Sutherland[2], Mohammad Rassool[3], Lindsay Zurba[4], Olena Ivanova[1], Salome Charalambous[5], Celso Khosa[6], Robert S. Wallis[5], Michael Hoelscher[1,7,8,9], Claire Calderwood[10], Brian Allwood[11], Noemi Castelletti[1,8, 9], and Andrea Rachow[1,7,9]

[1]*Institute of Infectious Diseases and Tropical Medicine, LMU University Hospital, LMU Munich, Germany*

[2]*Vaccines and Immunity Theme, Medical Research Council Unit The Gambia at LSHTM, Fajara, The Gambia*

[3]*Clinical HIV Research Unit (CHRU), Wits Health Consortium (WHC), Health Science Research Office (HSRO), Faculty of Health Sciences, University of Witwatersrand, Johannesburg, South Africa*

[4]*Education for Health Africa, Durban, South Africa*

[5]*The Aurum Institute, TB Research Group, Johannesburg, South Africa*

[6]*Instituto Nacional de Saúde, Marracuene, Mozambique*

[7]*German Centre for Infection Research (DZIF), Partner Site Munich, Munich, Germany*

[8]*Fraunhofer Institute, Immunology, Infection and Pandemic Research, Munich, Germany*

[9]*Helmholtz Zentrum München, German Research Center for Environmental Health (HMGU), Neuherberg, Germany*

[10]*Clinical Research Department, London School of Hygiene & Tropical Medicine, London, United Kingdom*

[11]*Division of Pulmonology, Department of Medicine, Stellenbosch University & Tygerberg Hospital, Cape Town, South Africa*

On behalf of the TB Sequel Consortium

Contents

1 Dataset Details 2

2 Model Development Details 4

3 Evaluation Details 4

4 Model Performance 5

5 Result Stability 6

6 Supplementary Evaluations 7

7 TB-Sequel Consortium 7

1 Dataset Details

1.1 Study Flowchart

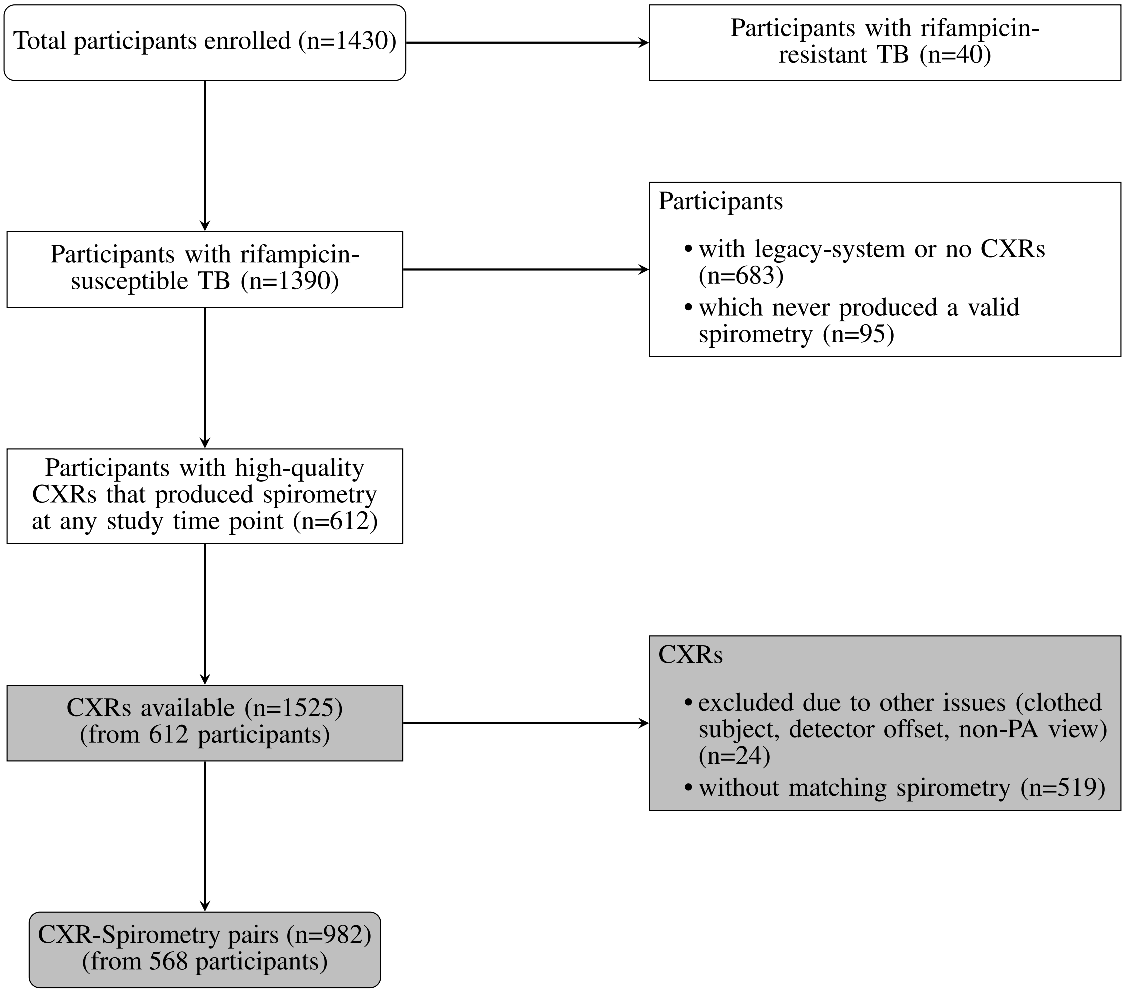

Supplementary Figure 1.1: Study flowchart

1.2 Sensitivity Analysis

To exclude the possibility of our estimation network to be purely informed by the peak flow, we performed a sensitivity analysis between peak expiratory flow rate and FEV1 as well as FVC shown in Supplementary Figure 1.2. The Pearson correlation coefficient was 0·688 and 0·511, respectively, which we argue is not strong enough for direct prediction. In comparison, the Pearson correlation coefficient between FEV1 and FVC was 0·881 despite various functional impairment types.

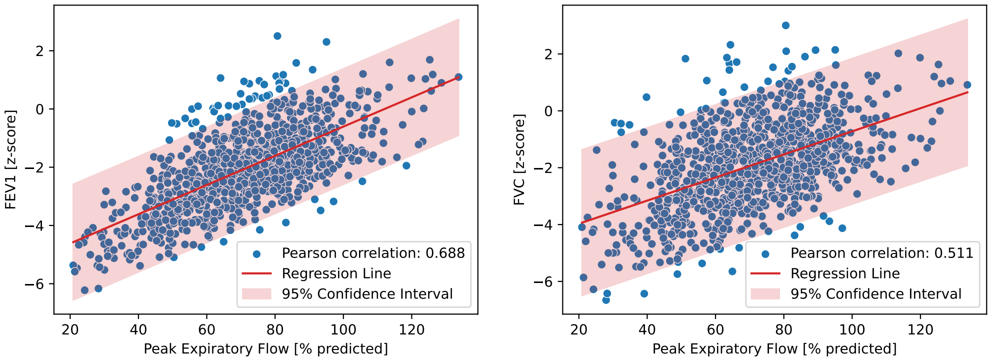

Supplementary Figure 1.2: Correlation between peak expiratory flow rate and FEV1 and FVC

1.3 Liters and z-scores

In our analysis, we show that estimating z-scores instead of liter values improves performance of our model though decoupling from demographic differences. To allow for a more intuitive understanding of the two and general orders of magnitude, we present a list of liters and z-scores for a mean member of our dataset in Supplementary Table 1.1.

Supplementary Table 1.1: Comparison between liters and z-scores for a mean member of our cohort (male, 169.7 cm tall, 35.4 years of age, *other* standard [SR1]). Liter values have been reduced from the predicted value (z-score 0.00) in step sizes of minimal clinical relevant change for FEV1 (0.23 liters) and FVC (0.33 liters) [SR2].

	liter value	z-score
FEV1	3.66	0.00
	3.43	-0.49
	3.21	-0.97
	2.98	-1.46
FVC	4.40	0.00
	4.07	-0.64
	3.75	-1.28
	3.42	-1.93

2 Model Development Details

All experiments were run on a Ubuntu 24.04.1 machine with a NVIDIA GeForce RTX 4070 Ti GPU using Python 3.11.10, torch 2.5.1, and pytorch-lightning 2.4.0. We use PyTorch's AdamW optimizer with a weight decay of 1e-5 and standard settings for the remaining parameters. We schedule the learning rate to reduce by a factor of 0.9 with a patience and cool-down of 2 up to a minimum value of $1e-7$. Hyperparameters used for training are shown in Supplementary Table 2.1. Each model takes about two hours to train.

Supplementary Table 2.1: Hyperparameter configuration of our models.

Parameter	Value
batch_size	16
dropout	0.1
epochs	250
feature_weights	densenet121-res224-all
fully connected estimator layers	[1025, 256, 256]
learning_rate	0.005
no_folds	9
weight_decay	1.0e-05

3 Evaluation Details

We combine the estimates of the networks trained as ensembles both naively by taking the mean and by using variance-weighted mean where applicable. Variance-weighted ensemble estimates are computed based on variance-weight w_i for each member i, adding a small constant ε for stability:

$$w_i = \frac{1}{\sigma_i^2 + \varepsilon}, \quad \varepsilon = 10^{-6}$$

Using the calculated weights, the weighted mean μ_w and the weighted tandard deviation σ_w are computed as:

$$\mu_w = \frac{\sum_{k=1}^{N} \mu_i \cdot \mathrm{w}_i}{\sum_{k=1}^{N} \mathrm{w}_i} \qquad \sigma_w = \sqrt{\frac{1}{\sum_{k=1}^{N} w_i}}$$

Given the combined cumulative distribution function $\Phi(x)$ of the standard normal distribution, we can compute the corresponding share of it falling inside and outside the decision corridor, the boundaries of which are given by τ_{lower} and τ_{upper}. With respect to the estimate being greater than the decision threshold, the resulting categories are:

$$p_{\text{negative}} = \Phi\left(\tau_{\text{lower}}\right)$$

$$p_{\text{positive}} = 1 - \Phi\left(\tau_{\text{upper}}\right)$$

$$p_{\text{uncertain}} = \Phi\left(\tau_{\text{upper}}\right) - p_{\text{negative}}$$

4 Model Performance

In Supplementary Figure 4.1, we show the Bland-Altman plot between estimates and measured values for the neural network presented in Table 2 estimating continuous z-scores from normalized CXRs and PEFR on the withheld test-set. In Supplementary Table 4.1, the effect of data representation and training paradigm on these values is reported. For comparison of liter values and z-scores please also refer to Supplementary Table 1.1.

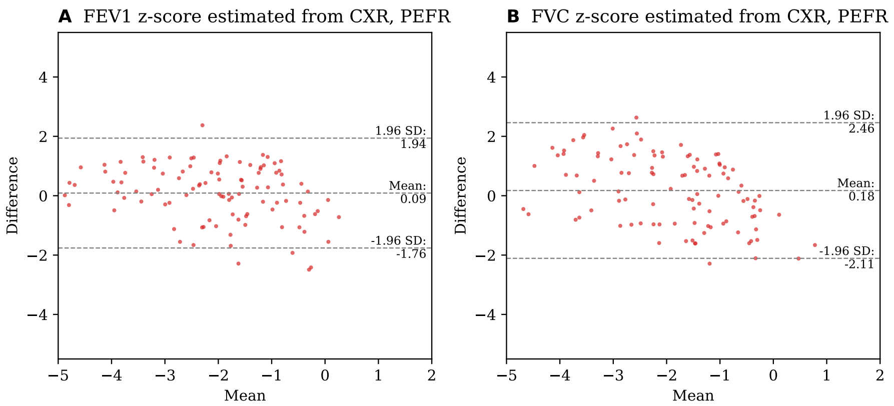

Supplementary Figure 4.1: Bland-Altman plots for model estimating z-scores from normalized CXRs and PEFR.

Supplementary Table 4.1: Bland-Altman mean of differences plus-minus 1.96 standard deviations over several data and training settings. We found the mean differences to be lowest for our probabilistic system without includsion of peak expiratory flow rate (PEFR). Only when augmenting with PEFR, root mean-squared error (RMSE) has a lower mean difference but a higher standard deviation of the mean differences. Huber loss has a lower mean difference than RMSE-trained models in the same cases, suggesting better fit for a deterministic system, but lacking the probabilistic component.

	**Network Input**	**Target**	**RMSE**	**Huber**	**NLL**
FEV1	CXR (raw)	liters	-0.364 ± 1.089	-0.124 ± 1.066	-0.117 ± 1.066
	CXR (norm)	liters	-0.409 ± 1.140	-0.158 ± 1.136	-0.135 ± 1.142
		z-score	0.292 ± 2.260	0.073 ± 2.212	0.011 ± 2.224
	CXR (norm) & PEFR	z-score	0.034 ± 1.894	0.077 ± 1.852	0.089 ± 1.850
FVC	CXR (raw)	liters	-0.470 ± 1.298	-0.143 ± 1.287	-0.147 ± 1.282
	CXR (norm)	liters	-0.510 ± 1.307	-0.185 ± 1.293	-0.177 ± 1.313
		z-score	0.305 ± 2.412	0.109 ± 2.365	0.073 ± 2.401
	CXR (norm) & PEFR	z-score	0.153 ± 2.330	0.137 ± 2.289	0.175 ± 2.285

5 Result Stability

To ensure our results are not based on one particular test split, we repeated all experiments with seven different random seeds. To avoid reporting extreme cases that may not generalize well and to improve robustness in our reporting, we excluded the best and worst performing seed by mean area under receiving operator characteristics for input data consisting of normalized CXRs augmented by peak expiratory flow-rate measures under negative log-likelihood training. Results are shown in Supplementary Table 5.1. We evaluate the relative performance of our proposed improvements in Supplementary Table 5.2 and ensure our network is not exploiting possible differences in data collection between sites in Supplementary Table 6.1.

Supplementary Table 5.1: Median [Q1-Q3] area under receiving operator characteristics for the stated network input and estimations targets for 9-fold cross-validated network ensemble trained on five random seeds.

	Network Input	Target	Samples Classified	RMSE	Huber	NLL
FEV1	CXR (raw)	liters	all	0.752 [0.751-0.791]	0.755 [0.748-0.764]	0.754 [0.735-0.785]
	CXR (norm)	liters	all	0.745 [0.742-0.788]	0.758 [0.738-0.808]	0.756 [0.722-0.805]
		z-score		0.819 [0.803-0.83]	0.822 [0.798-0.853]	0.831 [0.799-0.844]
	CXR (norm) & PEFR	z-score	all	0.903 [0.865-0.911]	0.904 [0.871-0.905]	0.896 [0.879-0.904]
			up to 10% uncertain			0.921 [0.894-0.937]
			up to 20% uncertain			0.944 [0.900-0.945]
FVC	CXR (raw)	liters	all	0.801 [0.754-0.839]	0.793 [0.739-0.798]	0.783 [0.735-0.821]
	CXR (norm)	liters	all	0.778 [0.729-0.783]	0.787 [0.732-0.789]	0.782 [0.721-0.793]
		z-score		0.838 [0.815-0.860]	0.843 [0.831-0.865]	0.838 [0.826-0.865]
	CXR (norm) & PEFR	z-score	all	0.839 [0.808-0.867]	0.850 [0.819-0.864]	0.853 [0.815-0.860]
			up to 10% uncertain			0.857 [0.824-0.870]
			up to 20% uncertain			0.875 [0.851-0.888]

Supplementary Table 5.2: Area under curve improvement of models across several seeds and 90% confidence interval by change of data representation under negative log-likelihood loss function.

	Change of Setup or Model	Δ AUC	90%CI
FEV1	raw data → normalized data (CXR, Spirometry)	+ 0.051	(0.017, 0.085)
	normalized data → addition of PEFR	+ 0.079	(0.037, 0.121)
	raw data → normalized and PEFR	+ 0.130	(0.091, 0.168)
FVC	raw data → normalized data (CXR, Spirometry)	+ 0.044	(0.018, 0.071)
	normalized data → addition of PEFR	+ 0.019	(-0.014, 0.053)
	raw data → normalized and PEFR	+ 0.063	(0.032, 0.095)

6 Supplementary Evaluations

Supplementary Table 6.1: Median [Q1-Q3] area under receiving operator characteristics of impairment classification accuracy by site. Results are thresholded with a band of 0.1 around the decision threshold and a required minimum CDF share of 0.8 outside that corridor since participants from the partner site in The Gambia generally have a worse lung-function and consequently more participants close to the decision threshold than those from South Africa (Table 1). As they are beyond the scope of this work, we refer the interested reader to [SR3] for further site-specific details.

Site	FEV1	FVC
The Gambia	0.891 [0.866, 0.942]	0.798 [0.795, 0.841]
South Africa	0.891 [0.857, 0.967]	0.794 [0.740, 0.861]

Supplementary Table 6.2: Median (Q1-Q3) area under receiving operator characteristics over time. Evaluated at diagnosis (M00), end of TB treatment (M06) and over the long-term follow-up of two or more years (M24+).

		M00		M06		M24+	
Overall	FEV1	n=13	0.96 (0.955-0.966)	n=50	0.926 (0.923-0.929)	n=40	0.846 (0.839-0.852)
	FVC		0.919 (0.909-0.93)		0.911 (0.908-0.914)		0.84 (0.834-0.846)
CXR Normal	FEV1	n=0	NA	n=8	0.719 (0.702-0.737)	n=25	0.887 (0.88-0.894)
	FVC		NA		NA		0.863 (0.854-0.871)
CXR Abormal	FEV1	n=13	0.96 (0.955-0.966)	n=42	0.93 (0.926-0.933)	n=14	0.706 (0.69-0.722)
	FVC		0.919 (0.909-0.93)		0.904 (0.9-0.908)		0.82 (0.808-0.831)
Female	FEV1	n=4	NA	n=15	1.0 (1.0-1.0)	n=12	1.0 (1.0-1.0)
	FVC		NA		1.0 (1.0-1.0)		0.805 (0.789-0.821)
Male	FEV1	n=9	0.909 (0.898-0.92)	n=35	0.883 (0.879-0.888)	n=28	0.846 (0.839-0.853)
	FVC		1.0 (1.0-1.0)		0.867 (0.862-0.873)		0.884 (0.878-0.891)
HIV Positive	FEV1	n=6	1.0 (1.0-1.0)	n=16	1.0 (1.0-1.0)	n=13	0.901 (0.893-0.91)
	FVC		0.674 (0.642-0.705)		1.0 (1.0-1.0)		0.911 (0.902-0.92)
HIV Negative	FEV1	n=7	0.639 (0.609-0.67)	n=34	0.864 (0.858-0.87)	n=27	0.827 (0.818-0.835)
	FVC		NA		0.835 (0.829-0.841)		0.802 (0.792-0.811)

7 TB-Sequel Consortium

Medical Research Council Unit The Gambia at LSHTM

Co - PIs: Beate Kampmann[2], Jayne Sutherland[2]
TB Lab: Basil Sambou[2], Abi-Janet Riley[2], Binta Sarr[2]
Immunology Lab: Caleb Muefong[2], Georgetta Daffeh[2]
Clinical Team: Olumuyiwa Owolabi[2], Shamanthi Jayasooriya[2], Abdou Sillah[2], Monica Davies[2], Alhaji Jobe[2], Momodou Jallow[2], Lamin Bah[2], Simon Badjie[2], Salieu Barry[2], Lamin Bah[2], Simon Badjie[2], Kairaba Kanyi[2], Abdoulie Tunkara[2], Gambia Sowe[2], Isatou Loum[2], Awa Touray[2], Mustapha Bah[2], Rohey Jallow[2], Simon Donkor[2]

Mbeya Medical Research Centre

PI: Nyanda Elias Ntinginya[12]
Co - PIs: Issa Sabi[12], Tina Minja[12]
TB Lab: Daniel Mapamba[12], Emmanuel Sichone[12]
Immunology Lab: Mkunde Chachage[12], Abisai Kisinda[12], Lwitiho Sudi[12]
Clinical Team: Elimina Siyame[12], Julieth M. Lalashowi[12]

University of Witwatersrand

PI: Ian Sanne[3]
Co - PI: Mohammed Rassool[3]
TB Lab: Lyndel Singh[3]
HE2RO: Denise Evans[3], Kamban Hirasen[3], Nelly Jinga[3]

Instituto Nacional de Saúde

PI: Ilesh Jani[6]
Co - PIs: Celso Khosa[6], Nilesh Bhatt[6]
TB Lab: Sofia Viegas[6], Carla Madeira[6], Khalide Azam[6], Cláudio Abujate[6], Narciso Macie[6], Tatiana Fernando[6]
Immunology and Safety Lab Lab: Nádia Sitoe[6], Salomão Manjate[6], Vânia Maphossa[6], Alberto Machaze[6]
Clinical Team: Cristovão Matusse[6], Antonio Machiana[6], Candido Azize[6], Alrindo Machava[6]
Socio-economic Team: Celina Nhamuave[6], Elvira Monteiro[6]

Institute of Infectious Diseases and Tropical Medicine, LMU University Hospital

Andrea Rachow[1], Michael Hoelscher[1], Olena Ivanova[1], Anna-Maria Mekota[1], Elmar Saathoff[1], Lin Luming[1], Olga Baranow[1], Abhishek Bakuli[1], Noemi Castelletti[1], Alberto Beyersdorff[1], Kathrin Held[1], Christof Geldmacher[1], Friedrich Riess[1], Fidelina Zekoll[1]

The Aurum Institute

Gavin Churchyard[5], Robert Wallis[5], Salome Charalambous[5], Kavindhran Velen[5], Farzana Sathar[5], Fadzai Munedzimwe[5]

Research Center Borstel

Stefan Niemann[13], Viola Dreyer[13], Ulrich Schaible[13], Christoph Leschczyk[13]

Associated Investigators

Knut Lönnroth[14], Lindsay Zurba[4]

[12]NIMR-Mbeya Medical Research Centre, Mbeya, United Republic of Tanzania
[13]Research Center Borstel, Leibniz-Center for Medicine & Biosciences, Borstel, Germany
[14]Karolinska Institutet, Stockholm, Sweden

Supplementary References

SR1. Quanjer P, Stanojevic S, Cole T, Baur X, Hall G, Culver B, Enright P, Hankinson J, Ip M, Zheng J, and ERS Global Lung Function Initiative JS on behalf of. Multi-ethnic reference values for spirometry for the 3-95-yr age range: the global lung function 2012 equations. Eur Respir Journal 2012 Dec; 40:1324–43

SR2. Herpel L, Kanner R, Lee S, Fessler H, Sciurba F, Connett J, and National Emphysema Treatment Trial Research Group RW on behalf of. Variability of spirometry in chronic obstructive pulmonary disease: results from two clinical trials. Am J Respir Crit Care Med 2006; 173:1106–13

SR3. Rachow A, Ivanova O, Bakuli A, Khosa C, PNhassengo, I Sabi OOSJNN amd, Rassool M, Bennet J, Niemann S, Mekota A, Allwood B, Wallis R, Charalambous S, Hoelscher M, and Churchyard G. Performance of spirometry assessment at TB diagnosis. Int J Tuberc Lung Dis 2023; 1:850–7
