# Supplementary material for: Deep learning approach for probabilistic pulmonary function estimation from chest X-ray and peak expiratory flow rate
Source: Commun Med (Lond). 2026 Jun 9;6:330. doi: 10.1038/s43856-026-01702-7 (PMC13249966; doi:10.1038/s43856-026-01702-7)
Supplement: Supplementary file 2 — Description of Additional Supplementary files [file 43856_2026_1702_MOESM2_ESM.docx]

**Description of Additional Supplementary Files**

File name: Supplementary Data 1-2

Description: Source data
